# What have we Learnt from the UK Triannual Case Reviews about the Role of Parental Mental Illness in Serious Abuse Related Harm of Young Children?

**DOI:** 10.1192/j.eurpsy.2022.94

**Published:** 2022-09-01

**Authors:** A. Wieck

**Affiliations:** 1 Greater Manchester Mental Health NHS Foundation Trust & University of Manchester, Psychiatry, LT, United Kingdom; 2 Greater Manchester Mental Health NHS Foundation Trust & University of Manchester, Psychiatry, Manchester, United Kingdom

**Keywords:** Abuse related harm, Parental mental illness, Infants, Children

## Abstract

Up to one in four young children have been estimated to be exposed to parental mental illness. Although the majority of affected parents will not harm their children, is is now well established that parental mental illness places children at an increased risk of all types of injury. Both maternal and paternal mental illness are implicated. Children of parents with mental illness are at the greatest excess risk of being injured in their first year of life, and this applies particularly to violence related harm. Within the first year, the risk is highest during the first three months after birth. A number of parental and family background factors have been identified in the UK triannual serious case reviews of abuse related harm to children and in other publications that can increase or decrease the risk. These findings have significant implications for clinical practice. In order to mitigate the risks to young children, an early assessment of a parent with a mental illlness, close collaboration between mental health, social care and other involved professionals, and a comprehensive care package which aims to improve parental mental health health, reduce other risk factors and exploit parental strengths and protective social factors, are essential. Mental health professionals have an important role to play in achieving improved outcomes for children at risk and their families.

**Disclosure:**

No significant relationships.

